# Concentrated growth factor therapy as cosmetic treatment in discoid lupus erythematosus

**DOI:** 10.1111/1346-8138.17114

**Published:** 2024-02-06

**Authors:** Xiao‐Shuang Yang, Ting‐Ting Wang, Yu‐Xin Ding, Yu‐Hong Chen, Zhong‐Fa Lv

**Affiliations:** ^1^ Department of Dermatology Second Affiliated Hospital, Zhejiang University School of Medicine Zhejiang Hangzhou China

**Keywords:** alopecia, concentrated growth factor, discoid lupus erythematosus, platelet‐rich ‐plasma

## Abstract

Discoid lupus erythematosus (DLE) is a disfigurement disease. The atrophic scar and hair loss of this disease are followed by cosmetic defects and profoundly impact psychological health. Concentrated growth factor (CGF) has been widely adopted in medical cosmetology. Here we report a 36‐year‐old female systemic lupus erythematosus patient with a 5‐year history of alopecia in DLE, who was recommended for CGF therapy and experienced hair regrowth. We suggest that CGF may be an effective cosmetic treatment for DLE.

## 1 INTRODUCTION

Up to 60% of discoid lupus erythematosus (DLE) patients have scalp involvement.^1^ The lymphocytic inflammatory process replaces the hair follicle units with fibrous tissue to destroy them, rendering conventional therapies ineffective. Concentrated growth factor (CGF), a novel autologous plasma extract, plays a distinct role in modulating the inflammatory response and fibroblast differentiation, inducing angiogenesis, skin regeneration, and even new hair follicle. Here, we reported a 36‐year‐old female with scarring alopecia secondary to DLE who experienced partial hair regrowth after treatment with intralesional CGF injections.

## 2 CASE DESCRIPTION

**FIGURE 1:**
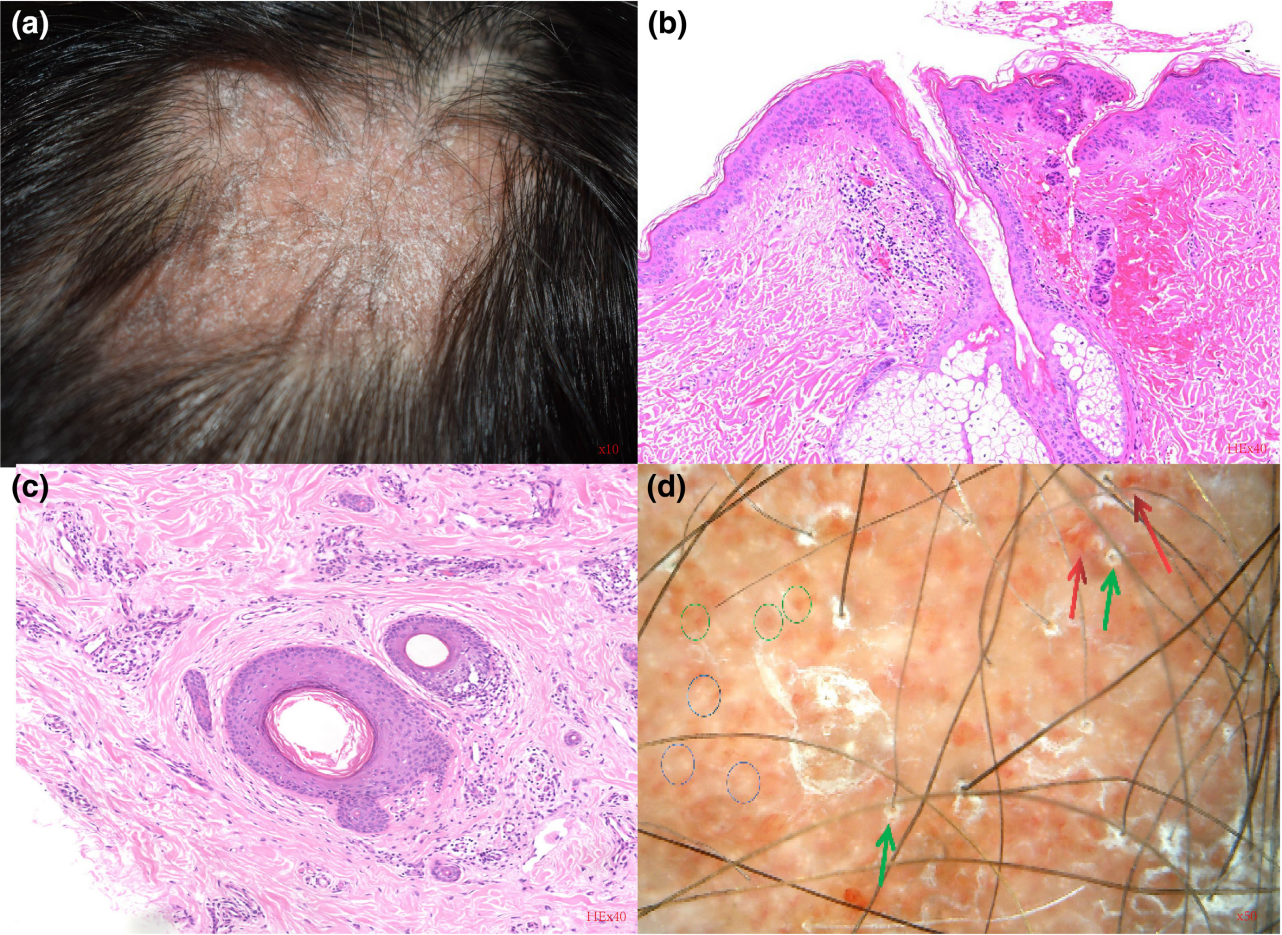
A 5 x 6‐cm patch of hair loss on the patient's vertex scalp and an erythematous, atrophic patch. (a) The lesion also included adherent scales on the surface (×10). (b, c) Biopsy from the lesional skin featuring dermo‐epidermal junction and hair follicles with mild vacuolar alteration of basal cells, inflammatory cells such as lymphocytes and plasma cells, infiltrating around the hair follicles and the blood vessels, and some fibrosis around the hair follicles (Hematoxylin‐Eosin stain ×40). (d) Dermoscopy showing pink‐white background, absent follicular openings, fibrotic white dots (blue circles), a few red dots (green circles), arborizing vessels (red arrows), follicular plugging (green arrows), and adherent scales (×50).

**FIGURE 2:**
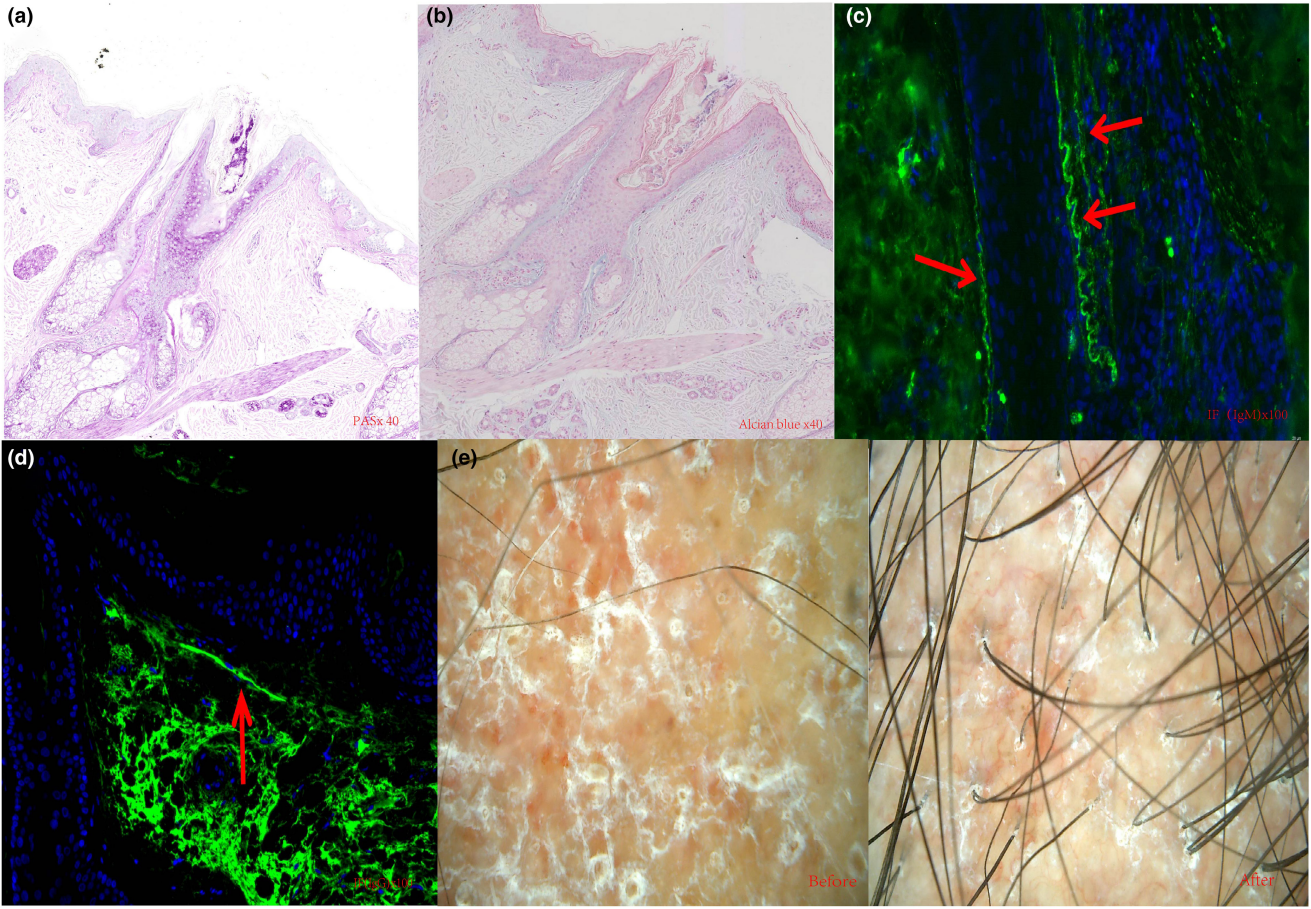
(a) Periodic acid‐Schiff stain showed mild thickening of the basement membrane (×40). (b) Diffuse deposition of mucin was observed by Alcian blue staining (×40). (c, d) Direct immunofluorescence photomicrograph showing granular deposition of IgM and IgG (light green) at the stroma epithelial junction of hair follicles. The nuclei of the cells are counterstained with 4′,6‐diamidino‐2‐phenylindole (light blue) (×100). (e) Before and after dermoscopy photographs of concentrated growth factor therapy in the central location of the skin lesion (×50).

**FIGURE 3:**
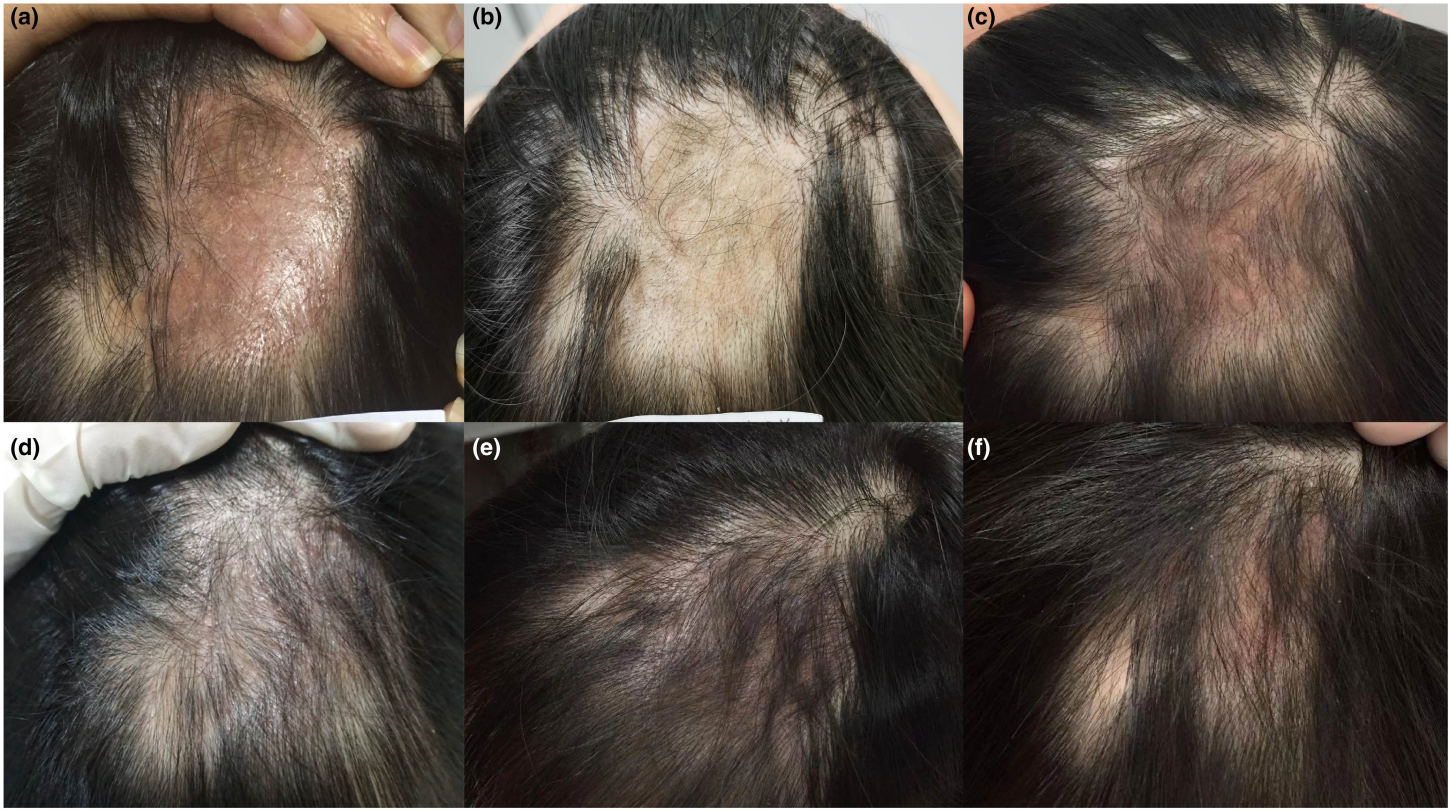
Effect of concentrated growth factor therapy for discoid lupus erythematosus alopecia: contrast clinical photographs before and after treatment once a month for six months showing partial new hair growth in alopecia lesion. (a) The first month, (b) the second month, (c) the third month, (d) the forth month, (e) the fifth month, and (f) the sixth month.

A 36‐year‐old female with a 20‐year history of systemic lupus erythematosus (SLE) was referred to our clinic with the complaint of progressive scalp alopecia. She complained that patchy hair loss on the vertex scalp with pruritus had affected her appearance for 5 years, during which time the alopecia area had continuously extended from the size of a coin to a patch. A 5 × 6‐cm patch of hair loss and an erythematous, atrophic patch were revealed on her vertex scalp in physical examination, accompanied by adherent scales on the surface (Figure 1a). She was in good health and denied any other autoimmune diseases. The results of dermoscopy indicated a pink‐white background, absence of follicular openings, fibrotic white dots, a few red dots, arborizing vessels, follicular plugging, and adherent scales (Figure 1d). The laboratory results demonstrated antinuclear antibody titer 1:80, negative anti‐double‐stranded DNA, normal anti‐Immunoglobulin M (IgM) and Immunoglobulin G (IgG) antibodies, and normal serum complements C3 and C4. The biopsy from lesioned skin showed the dermo‐epidermal junction and hair follicles with mild vacuolar alteration of basal cells, inflammatory cells such as lymphocytes and plasma cells infiltrating around the hair follicles and the blood vessels, and some fibrosis around the hair follicles (Figure 1b,c). Periodic acid‐Schiff stain showed mild thickening of the basement membrane and mucin was observed by Alcian blue staining (Figure 2a,b). Direct immunofluorescence photomicrography showed granular deposition of IgM and IgG (light green) at the stromal‐epithelial junction of hair follicles (Figure 2c,d). She was diagnosed with scarring alopecia secondary to DLE. Under the stable condition of SLE, medrol and hydroxychloroquine (5 and 200 mg, respectively) were given once daily as the maintenance therapy. The erythema and pruritus were relieved after topical or intralesional corticosteroids and long‐time oral hydroxychloroquine therapy, but there was no hair regrowth. Afterwards, local subcutaneous injection once a month for 6 months of autologous CGF to the alopecia lesion was recommended. The detailed CGF therapy procedure for each treatment was as follows: 9 mL of autologous venous blood was collected into a sterile vacuette tube and then immediately centrifuged using a variable speed centrifuge (preset 4°C conditions; Medifuge MF200, Silfradent srl, Forli, Italy). The procedure was as follows for about 12 min: acceleration for 30 s, 408 *g* for 2 min, 323 *g* for 4 min, 408 *g* for 2 min, 503 *g* for 3 min, and finally deceleration for about 36 s. A total volume of approximately 2.5 mL of CGF was obtained from the middle layer and subcutaneous injections were performed with a 30‐gauge needle at 0.2 mL/cm^2^. The patient reported prominent hair regrowth after just two injections within 3 months and eventually exhibited increased terminal hair regrowth in the alopecia area. No other adverse events were reported except for pain at the injection site. Representative photographs of the patient before and after using the CGF are presented in Figures 2e and 3a–f.

## 3 DISCUSSION

Scalp involvement is reported in up to 60% of patients with DLE, with immune dysregulation, dermatism, vascular destruction, and fibrosis.^1^, ^2^ CGF covers more growth factors, including platelet‐derived growth factor (PDGF), transforming growth factor β1 (TGF‐β1), vascular endothelial growth factor (VEGF), and a unique CD34 cell population, which has been identified to play a distinct role in angiogenesis, wound healing, tissue repair, and regeneration.^3^ CGF therapy is rarely mentioned in related reports on DLE alopecia. Huijuan Fang et al. reported that a 30‐year‐old woman with DLE alopecia administered CGF combined with corticosteroids experienced approximately 90% of hair regrowth and erythema disappeared after 3 months of treatment.^4^ Hannah Polster et al. demonstrated significant hair regrowth in areas of cicatricial alopecia in a female patient with DLE alopecia after receiving PRP therapy every 3 months who had previously experienced SLE.^5^

PRP plays a positive role in alleviating autoimmune diseases such as scleroderma, pemphigus vulgaris, and alopecia areata.^6^, ^7^ CGF can regulate the macrophage‐mediated immune response, in which the TGF‐β1 is acknowledged to inhibit the proliferation and function of lymphocytes as well as the function of macrophages.^8^ The DLE lesions are accompanied by destruction of the vessel wall. CGF‐derived cells (CD34+ cells) release TGF‐β1, VEGF, and matrix metalloproteinases, which could induce endothelial cell proliferation, migration, and tubular structure formation.^9^ At present, there is no representative basic research on CGF improvement in fibrosis, but studies have demonstrated that PRP negatively modulated fibroblast differentiation.^10^, ^11^ Cases of PRP therapy in cicatricial alopecia have also been reported, referring to lichen planopillaris, central centrifugal cicatricial alopecia, and frontal fibrosing alopecia.^9^ Whether or not the anti‐fibrosis mechanism is consistent with PRP still needs further study. CGF studies in different types of stem cells have found more proliferation and differentiation of stell cells using CGF.^12^ PRP not only promotes the proliferation of dermal papilla cells and hair follicle stem cells, but also decreases apoptosis.^13^ The abundant growth factors in CGF or PRP such as insulin like growth factor I, VEGF, and PDGF, may promote the proliferation and differentiation of hair bulge stem cells and induce epidermal hyperplasia.^12^, ^14^

The appearance of hair regeneration in the present case also confirmed the anti‐inflammatory and skin tissue regeneration capability of CGF. More studies are required to evaluate the efficacy of CGF in treating DLE.

## CONFLICT OF INTEREST STATEMENT

None declared.

## Data Availability

The data used during the current study are available from the corresponding author upon reasonable request. Patient consent and the approval of the Ethics Committee of Second Affiliated Hospital, Zhejiang University School of Medicine (HangZhou, China) (2020/012) were obtained before the CGF therapy.
